# Analysis of Selected Endocrine Disrupters Fraction Including Bisphenols Extracted from Daily Products, Food Packaging and Treated Wastewater Using Optimized Solid-Phase Extraction and Temperature-Dependent Inclusion Chromatography

**DOI:** 10.3390/molecules24071285

**Published:** 2019-04-02

**Authors:** Aleksandra Kaleniecka, Paweł K. Zarzycki

**Affiliations:** Department of Environmental Technologies and Bioanalytics, Faculty of Civil Engineering, Environmental and Geodetic Sciences, Koszalin University of Technology, Śniadeckich 2, 75-453 Koszalin, Poland; a.kaleniecka@gmail.com

**Keywords:** cyclodextrin, bisphenols, endocrine disrupters, micropollutants, daily use products, sewage water, supramolecular complex, encapsulation, temperature effects

## Abstract

The aim of this research is to demonstrate the concept and ability for the fast and preliminary screening of complex food and environmental samples for the presence of endocrine disrupters fractions, consisting of low-molecular mass micropollutants, particularly various bisphenols (A, B, C, E, F, S, Z, AF, AP, BP and FL). The developed analytical protocol for this research requires two main steps: (i) optimized solid phase extraction (SPE) for selective isolation, purification and pre-concentration of target fraction, and (ii) selective temperature-dependent inclusion chromatography for samples analysis via a HPLC-UV-VisDAD system using isocratic elution and internal standard quantification approach. The chromatographic experiment revealed that both β-CD and its hydroxypropyl derivative strongly interact with selected bisphenols. This is in contrast to the steroids and PAHs molecules investigated previously, where a strong interaction with β-cyclodextrin was observed. Integrated areas derived from acquired chromatographic profiles for each individual sample were used as the simple classification variable enabling samples comparison. We demonstrated that the proposed analytical protocol allows for fast estimation of EDC fractions in various daily use products, food and environmental samples. The materials of interest were selected due to the presence in surface water ecosystems of their residues, and finally, in raw wastewater including rice bags, plastic bags, cloths, sanitary towels, fish baits and various plastic foils from food products. Treated sewage water released directly to the environment from a municipal treatment plant (Jamno, Koszalin) was also investigated. It has been demonstrated that a whole range of low-molecular mass compounds, which may be detected using UV-Vis detector, can easily be emitted from various in daily use products. The presence of micropollutants in treated wastewater, water ecosystems and plastic waste utilization via technological wastewater treatment processes must be addressed, especially in terms of microplastic-based pollutants acting as endocrine disrupters. It is hoped that the proposed simple analytical protocol will be useful for fast sample classification or selection prior to advanced targeted analysis involving the more accurate quantification of specific analytes using e.g., mass spectrometry detectors.

## 1. Introduction

Hormonal systems are considered essential elements of all living organisms. Many biogenic or synthetic chemicals that are present in our natural environment may affect hormone receptors and modulate hormone actions, as well as significantly changing their transport within multi-cellular organisms [1]. It should be noted that the problem of endocrine disrupting phenomenon was identified and brought to the attention of the scientific community in the 1980s. At that time, deformities in fish were observed in the European rivers [2]. Presently, endocrine modulation is mainly related to potentially dangerous consequences for humans and wildlife, due to the presence of natural and artificially-generated anthropogenic endocrine disrupting compounds (EDCs), mainly in the aquatic environment [3]. One important issue is that EDCs are not defined by their chemical nature, but by their biological effect [4]. Consequently, many different classes of micropollutants, including pesticides, polycyclic aromatic hydrocarbons, plasticizers, polychlorinated biphenyls, dioxins as well as natural steroids like phytoestrogens, can be collectively referred as EDCs [5].

One of the important groups of chemicals regarded as EDCs is PAH derivatives and bisphenols. PAHs may be considered endocrine disrupters due to the fact that their structures can be present in several commonly-consumed drugs like propranolol and its derivatives [6]. Bisphenols belong to a fairly homogenous group of low-molecular mass compounds, which, with some exceptions (e.g., bisphenol S), are based on diphenylmethane skeleton (Figure S1; Supporting Information). This group of low-molecular mass chemicals, particularly bisphenol A, may mimic the action of natural steroids, mainly estrogens. It should be noted that bisphenols have been classified as “pseudo-persistent” chemicals [7]. Bisphenols are commonly applied on an industrial scale as chemical agents mixed with raw polymers resulting in soft plastic materials. As a consequence of the global plastic waste problem, various low-molecular mass plasticizers can be found in all water ecosystems on the Earth [8]. Endocrine disruption is commonly considered to be a serious global issue, and so far, there is no practical solution to this problem, particularly considering the common wastewater treatment technologies which are applied to the organic waste generated by large cities or industrial areas. It is very important to keep in mind that bisphenols and other EDC micropollutants like steroids (particularly estrogens) are very stable under typical environmental conditions, and their concentration ranges from ng to μg levels per liter, depending on water type [9]. Therefore, the monitoring of these low-molecular mass micropollutants in aquatic environments is of great importance. For that reason, numerous studies have been conducted to develop analytical procedures which are suitable for the quantification of a wide range of steroids in water samples, based on various analytical approaches as well as sample concentration and quantification concepts [10,11,12,13,14,15].

Wastewater treatment can be classified as a technological process enabling the removal of pollutants and sediments from sewage. During this process, dissolved chemical substances, colloids and suspensions may be also removed. Sewage treatment plants can remove a very wide range of organic substances. Importantly, while solid particles, carbonaceous substances, nutrients and pathogens are effectively eliminated from wastewater, the removal of micropollutants is usually inadequate. Usually, common municipal treatment plants are not appropriately optimized for the removal of low-molecular mass micropollutants and their metabolites through mechanical and biological processes [16,17,18,19]. Therefore, it is necessary to improve technological processes for the efficient removal of potentially unsafe micropollutants from raw or purified wastewater [20]. Additionally, there is growing interest regarding the development of green technologies invented for the efficient removal of low-molecular mass compounds, referred to as micropollutants, which persist in food and agricultural products and water ecosystems, and which are generated during technological wastewater treatment processes. This interest is the consequence of an extensive research performed over the last decade focusing on the determination of such micropollutants in environmental ecosystems and within living organisms, as well as studies considering the potential risk of long-term exposure to these molecules for both animal and human health [21].

It should be emphasized that endocrine disruption is commonly considered to be a serious global issue, and so far, there is no practical solution to this problem, particularly if we consider common wastewater treatment technologies applied to organic waste which is generated by large cities or industrial areas. Most recently, several studies have strongly indicated that the efficient elimination of a number of micropollutants, for example, heavy metals or organic compounds including benzene derivatives, polycyclic aromatic hydrocarbons and steroids, can be performed using host-guest complexation involving cyclodextrin-based materials. This technology can be applied to sewage water technological processes, mainly through the solid/polymeric adsorbents [22,23].

The aim of this research is to demonstrate the concept and ability for predominantly non-targeted screening of complex food and environmental samples toward presence of EDCs micropollutants fraction, with polarity ranging from estetrol to progesterone. The developed analytical protocol for this research consists of two main steps: (i) optimized solid phase extraction (SPE) for selective isolation, purification and preconcentration of target fraction and (ii) temperature-dependent inclusion chromatography for samples analysis via isocratic HPLC-UV-VisDAD system using the internal standard quantification approach. Integrated areas derived from acquired chromatographic profiles for each individual sample were then used as the classification variable enabling sample comparisons. We demonstrated that the proposed analytical protocol allows for fast estimation of non-targeted EDC fractions in various daily use products, food and environmental samples. The materials of interest were selected due to the presence of their residues in surface water ecosystems, and finally, in raw wastewater including rice bags, plastic bags, cloths, sanitary towels, fish baits and various plastic foils from food products. Treated sewage water released directly to the environment form a municipal treatment plant (Jamno, Koszalin) was also investigated. It is hoped that proposed simple analytical protocol will be useful for fast sample classification or selection prior advanced targeted analysis involving more accurate quantification of specific analytes using e.g., mass spectrometry detectors.

## 2. Results and Discussion

### 2.1. Optimization of Bisphenols Separation and Selected Validation Issues of the Quantification Protocol

This part of the research reports a new analytical protocol enabling the rapid separation of eleven bisphenols using temperature-dependent inclusion chromatography (based on HPLC system) involving mobile phases modified with natural and biodegradable biomaterials: cyclodextrins. The separation process was performed on a typical octadecylsilane (low carbon load C18) analytical column [24,25]. In particular, the retention of supramolecular host-guest complexes occurring between native β-cyclodextrin (β-CD) or its highly soluble in water (and water/organic liquids) hydroxypropyl derivative (2-HP-β-CD) and target compounds, namely: bisphenol A, B, BP, Z, AF, AP, C, E, F, FL and S, was analyzed (Figure S1; Supporting Information). It has been documented that temperature-sensitive inclusion complexes created within the chromatographic mobile phase enable multiple separation of target molecules in a fast and optimal manner. Optimization results concerning total retention time, peaks distribution on chromatograms and peaks resolution were compared to chromatographic behavior of analytes under plain binary mobile phase (acetonitrile/water 35% *v*/*v*) conditions. Similar to previously reported research on polycyclic aromatic hydrocarbons and steroid hormones mixtures, the results of the present study have revealed strong interaction of cyclodextrins with analytes, particularly in the subambient temperature region [12,26,27,28,29]. The raw retention data set obtained under subambient, room and elevated temperatures conditions (10, 20, 30, 40 and 50 °C) is presented in Table 1. As with steroids molecules analyzed in our previous studies, it has been found that the relationship between the logarithmic form of the chromatographic retention factor (*k*) and reversed temperature (1/T [K]) is in most cases non-linear (with the exception of bisphenol S). Table 2 consists of calculated values of quadratic regression coefficients (a, b, c) and determination coefficient (*r*^2^) for the equation in the form of Equation (1):

ln*k* = a(1000/T)^2^ + b(1000/T) + c (1)

Considering the determination coefficient (*r*^2^) values presented in Table 2, an aquadratic model can be appropriate for determining the given analyte retention within temperatures investigated. These data were necessary to calculate optimization parameters for multiple separations of bisphenols and an internal standard mixture, particularly: total analysis time (*t_max,min_*), resolution (*R_s,min_*) and peaks distribution along the time axis (*r*; relative resolution product). The last optimization parameter was calculated according to Equation (2):

*r* = Π*R*_*s* i+1_/[Σ*R*_*s* i+1_)/(n − 1)]^n−1^ (2)

**Figure 1:**
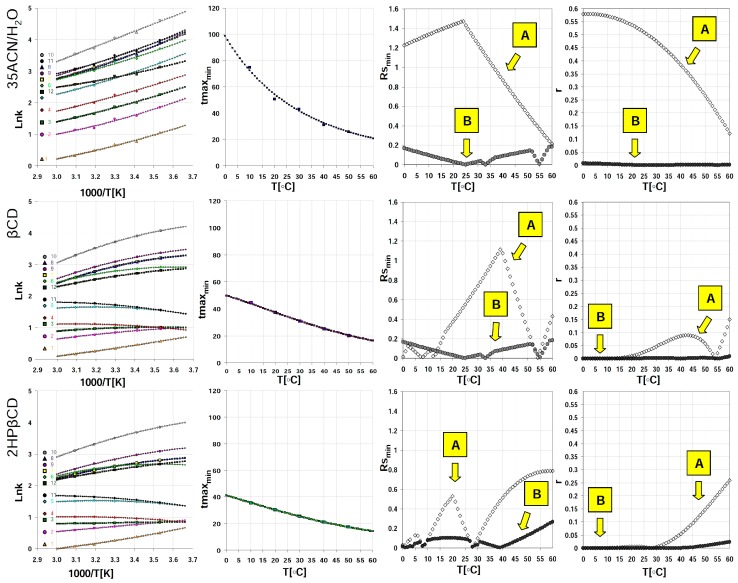
Raw chromatographic data and optimization graph concerning retention of studied bisphenols in terms of analysis time (*t_max,min_*), resolution (*R_s,min_*) and peaks distribution along time axis relative resolution product (*r*). **A**—tested bisphenol without bisphenol AF and bisphenol BP; **B**—all tested bisphenol.

**Figure 2:**
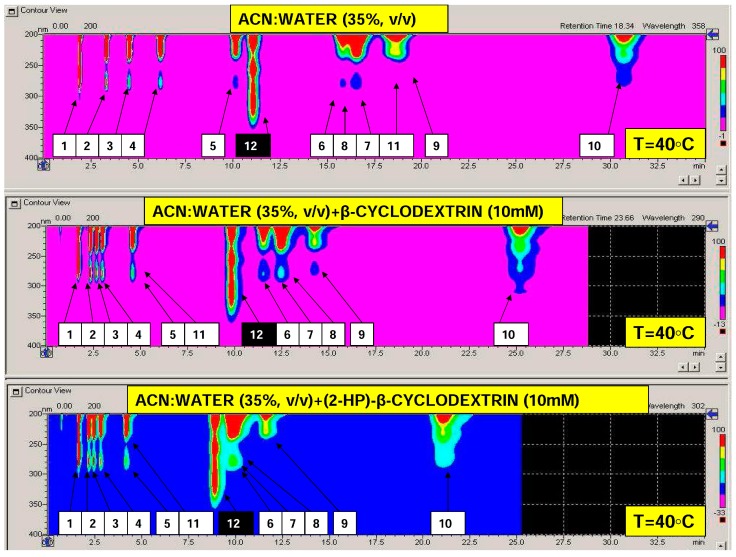
Isocratic separation of 12 substances (11 bisphenols and internal standard; analytes IDs according to data presented in Table 1A performed on 10 cm LC-18 column at temperature of 40 °C using plain binary mobile phase (top) and modified with different cyclodextrins at concentration of 10 mM (middle and bottom).

The approximate elution times of analytes, within temperatures ranging from 0 to 60 °C (with step 1 °C) together with the calculated optimization parameters profiles are visualized in Figure 1. With reference to total analysis time and considering the whole set of target analytes, elevated temperature region for the efficient separation of bisphenols may be preferred. Examples of isocratic separation performed at 40 °C and using different mobile phase additives are presented in Figure 2. As can be seen, baseline separation of selected bisphenols can be obtained and the retention time reduced using cyclodextrin additives. However, for efficient separation of the given bisphenols mixtures e.g., containing bisphenol Z (No 11), a subambient temperature region should be selected, if cyclodextrin modified phases are applied. In such cases, the total analysis time can be significantly reduced in comparison with plain acetonitrile–water mobile phase. It should be highlighted that using the isocratic systems studied, bisphenol AP (No 7) and bisphenol BP (No 8) cannot be separated, regardless of the mobile phase additive and temperature (Figure 1 and Figure 2). For such components of interest, a different concentration of cyclodextrins, macrocyclic additive type, acetonitrile % or gradient elution system should be tested and applied.

To illustrate and compare the efficiency of bisphenols interaction with macrocyclic additives the ratio *k*_0mMCD_/*k*_10mMCD_ for each temperature point was calculated (Table 3). As it was observed for different classes of low-molecular mass compounds, a strong interaction with macrocyclic additives at low temperatures is more significant [28,29]. A decrease in the retention time of analytes at a subambient temperature is particularly visible for bisphenol B (**5**) and Z (**11**) in the case of native cyclodextrin as well as for A (**4**), B (**5**), and Z (**11**) in case of a hydroxypropyl derivative. Such results suggest preferable interactions of cyclodextrins with bisphenols containing *n*-alkanes chains or saturated rings (cyclohexane). This observation could be useful for designing an selective chromatographic system for bisphenols analysis as well as the removal of such molecules from the liquid phase based on host-guest interaction.

Interestingly, the interaction intensity of bisphenols with the macrocycles investigated is similar for both: native β-CD and the hydroxypropyl derivative. In the case of low-molecular mass compounds, which strongly interact with β-CD, for example: steroids (17β-estradiol, testosterone, 20α-hydroxyprogesterone, diethylstilbestrol) and PAHs (1,8-dimethylnaphthalene, acenaphthenol and acenaphthylene), the observed interaction with hydroxypropyl derivative was less significant [11,27]. This can be applied for the selective analysis and/or removal of bisphenols using hydroxypropyl β-CD complexation systems.

Detailed validation of the quantification protocol (including detection limits, selectivity, intra/interday precision, method robustness and more) for various analytes including PAHs, steroids and bisphenol A using an internal standard substance (7,8-dimethoxyflavone) and temperature-dependent inclusion chromatography were reported previously [12,26]. In this work similar SPE extraction, separation and detection protocols were applied, therefore, in the case of the bisphenols group investigated only recovery studies were conducted. This is because the individual breakthrough curves for these compounds were not investigated and therefore, a previously optimized SPE protocol (for analytes polarities ranging from estetrol to progesterone) may have a significant impact on bisphenols determination. As can be seen from the recovery data presented in Table 4 the recovery rate of the internal standard substance (7,8-dimethoxyflavone) is high (94.35 ± 6.96), similarly to our previous studies involving different environmental and biological matrices [11,12,26]. In case of target bisfenols the recovery levels may be acceptable for preliminary screening studies for the majority of target analytes with the exception of bisphenols S, A and AF. It should be noted that in case of quantitative approach based on internal standard substance, the low recovery is not really a problem if the analytical signals of both internal standard peak and target analyte are strong enough to be visible above the baseline noise and meet the limit of detection (LOD) and limit of quantitation (LOQ) requirements. Considering the quantification criterion CV%, which should be equal 10 or less, determination of BPAP+BPBP as well as BPAF (CV%=15.87 and 12.24, respectively) can be treated as semi-quantitative. In the case of bisphenol S relatively low recovery was observed. This may affect the sensitivity of the quantification protocol for this molecule. However, appropriate optimization of the elution mixture (for SPE step) should improve the low recovery rate of this compound. High recovery of bisphenol A is associated with high background contents of this substance in distilled water from our laboratory. Therefore, in such cases the excess of bisphenol A can only be determined above the 100 µg/L level. High recovery of bisphenol AF can be associated with low chromatographic resolution and co-elution of matrix interfering peaks using short 10 cm analytical column.

### 2.2. Real Samples Analysis: Daily Products, Packaging and Treated Wastewater

There are a number of publications dealing with the quantification of bisphenol A and related endocrine disrupting micropollutants in various environmental matrices including water, soil and sediments. Especially, the problem of such low-molecular mass compounds is currently extensively investigated in terms of microplastic presence in water and in the tissues of living organisms as well as in relation to the potential endocrine modulation risk [30,31,32,33,34,35,36,37]. This is a consequence of a global environmental pollution with: (i) plastic originated macro objects (various plastic bags, containers, cosmetic sticks) that are slowly ground and disintegrated, mainly under marine conditions and (ii) the common modification of daily used products with plastic micro beads (e.g., present in cosmetics) [30,38,39]. However, interpretation of quantitative data reported in literature may be difficult, due to complex analytical matrices and a lack of widely acceptable standardized analytical protocols enabling quantification of multiple target compounds. Some of them are based on sensitive sensors [40,41,42,43,44,45], but this technology is still problematic due to the relatively low selectivity of such systems, especially in the case of target components with similar chemical structures. The main advantages of such an approach are the low cost of determination and a rapid quantification procedure. More time consuming and expensive but robust and widely accepted quantification protocols usually involve classical and miniaturized separation techniques (capillary electrophoresis, gas chromatography, liquid column/planar chromatography [46,47,48,49,50,51]. It should be noted that even if very efficient separation systems (e.g., multidimensional elution) and selective detectors are applied (based on fluorimetry, mass spectrometry or electrochemical detection), sample pretreatment is still needed (typically using SPE or SPME), which may strongly affect the quantification results [52].

This part of the study focus on the screening of low-molecular mass compounds that can be emitted from various daily products or which are present in treated wastewater. Chromatographic profiles of SPE extracts were recorded by UV-Vis DAD detector. Additionally, profiles of distilled and tap water samples obtained from our laboratory were analyzed. The extraction and separation protocol was optimized for the selective analysis of a wide range of matrix compounds (polarity from estetrol to progesterone) [13,26,53].

Additionally, the HPLC separation step involved selective interaction of target compounds with β-cyclodextrin in the mobile phase. As it was proven above, cleaning and elution solvents, which were selected and previously optimized for the quantification of various steroids, may also effectively clean and concentrate target bisphenols. Therefore, recorded HPLC profiles of SPE extracts should reflect the contents of known and unknown low-molecular mass compounds, which may work as endocrine disrupters, including bisphenols and steroids.

Selection of materials of interest was based on the observation that raw (non-treated) sewage water may be composed of various solid objects identified as e.g., plastic bags, cloths, sanitary towels, wet wipes and similar. Moreover, some food products are prepared by the boiling of grain portions in plastic bags (e.g., rice, buckwheat or wheat products), therefore, plastic decomposition products, which may be generated at elevated temperatures, can be finally present in raw sewage water. For this research, number of materials were investigated as potential sources of bisphenols fractions in sewage water. They are listed in Table 5. Plastic fishing bait was also investigated due to the potential problem of plasticizers emission from such products. This issue may be a problem in the lakes with strong anthropogenic pressure from the angling community.

The samples were processed with a 15 min boiling step in accordance with common food products preparation instruction (rice grains in plastic bags). In the case of the remaining samples the boiling step was performed to simulate a long term extraction process in the aquatic environment, similarly to e.g., food products stability tests that are conducted at elevated temperature [54,55]. In the case of tap water and sewage water, two protocols were applied (boiled and non boiled samples) to monitor the the effect of temperature on bisphenol A contents and to detect overall changes in chromatographic profiles during sample heating. All samples were processed using an SPE protocol and analyzed by HPLC DAD-UV-Vis separation/detection system.

**Figure 3:**
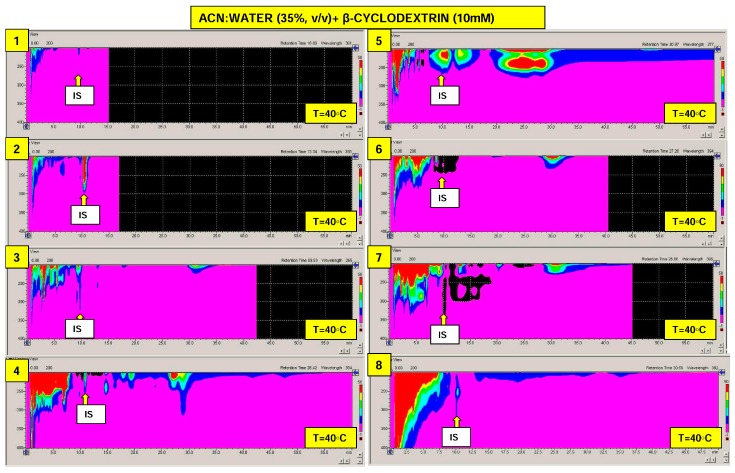

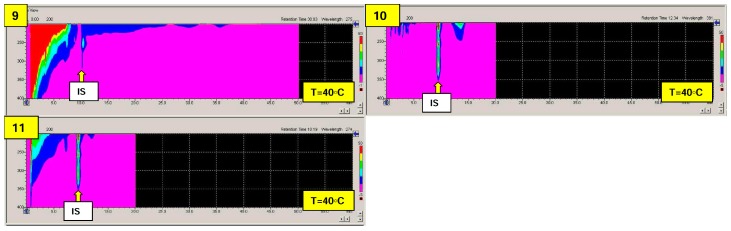
Typical DAD-UV-Vis chromatograms’ of samples investigated. Chromatograms labels: **1**. Boiledtap water, **2**. Rice bag, **3**. Plastic bag, **4**. Cloth, **5**. Fish baits, **6**. Sanitary towels, **7**. Wet wipes, **8**. Boiled purified sewage, **9**. Raw purified sewage, **10**. Distilled water, **11**. Raw tap water.

The results of this investigation are presented in the form of the diode array chromatograms presented in Figure 3. As can be seen, there are significant differences between all samples which underwent chromatography. These analyses clearly indicate that the products investigated can decompose to a number of low-molecular mass chemicals. Chromatographic profiles related to the analytical wavelength 280 nm (characteristic for bisphenols and phenolic compounds e.g., estrogenic steroids), are presented in Figure S2 (Supporting Information). These clearly indicate the massive differences in bisphenol A level that can be present in the extracts, based on quantification methodology involving internal standard addition. It has been found that the bisphenol A levels in these samples may change by 4 factors: from 0.1 to 107 (BA/IS ratio), which correspond to concentrations of this micropollutant from 5 to 4466 µg/L (Table 6). This observation is in agreement with data reported in literature, where extremely high contents of bisphenol A were found to be present in e.g., atmosphere (up to ng/m^3^ level), surface/ground water ecosystems (varying from ng to mg/L), food and drinking water (up to μg/L level), various paper based products (up to 26 mg/g) or food containers (μg amount per one plastic coated can or bottle) [56,57,58,59,60,61]. Unfortunately, the chromatogram complexity and detection type disable accurate identification/quantification of the remaining bisphenols. Therefore, in the future studies this problem should be solved by using a more efficient analytical column (25 cm long instead of the 10 cm that was used for the present study) and the application of a selective MS detection system.

**Figure 4:**
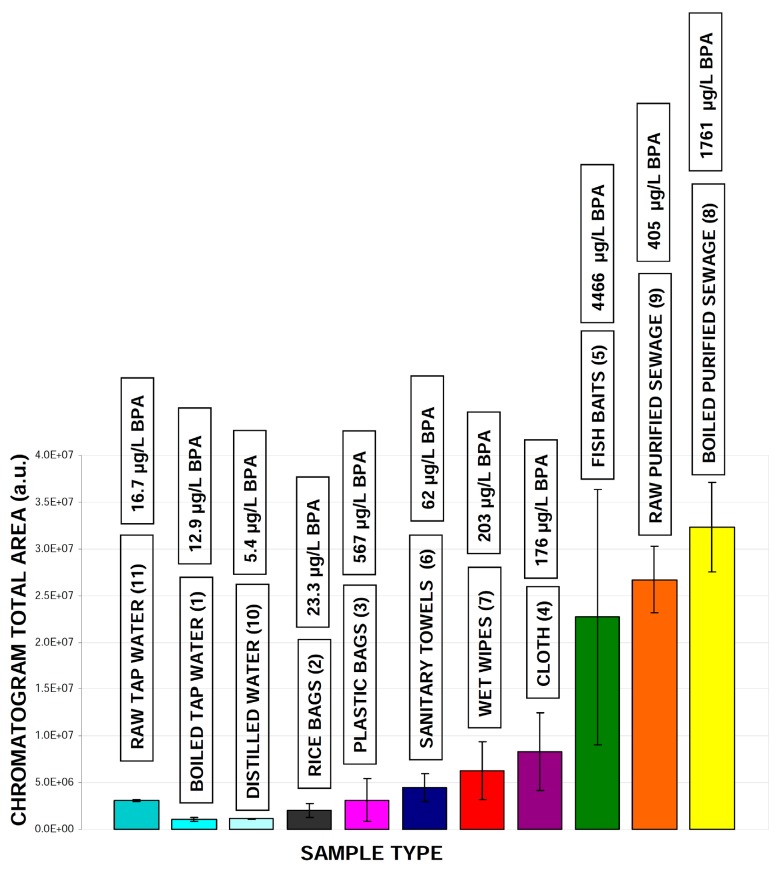
Integrated areas of all detected peaks on chromatograms (detection at 280 nm) calculated for various samples, which were prepared accordingly to our analytical protocol. Boxes above each material type indicate the calculated value of bisphenol A detected within each sample. Samples labeling: raw distilled water (**10**), raw tap water (**11**), boiled tap water (**1**), rice bags (**2**), various plastic bags (**3**), plastic fishing baits (**5**), various dust cloth (**4**), sanitary towels (**6**), raw purified sewage (**9**) boiled purified sewage (**8**) wet wipes (**7**). Numbers in parentheses are related to chromatograms numbers presented in Figure 3 and Figure S2.

Data presented on the chromatograms (Figure S2; Supporting Information) are visualized as the values corresponding to all peaks areas integrated for the given sample type (Figure 4). According to this data, there are significant differences in the total organic matrix emitted from the samples investigated. Heating of the sewage water does not change the overall contents of the SPE extracts, indicating that temperature manipulation cannot be really used to decrease of the level of low-molecular mass compounds level in treated sewage water. Interestingly, the level of bisphenol A was increased after the boiling process.

**Figure 5:**
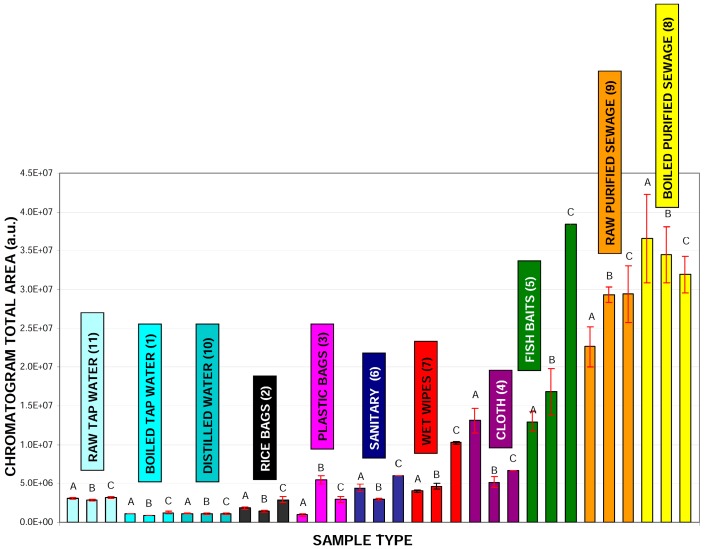
Integrated areas of all individual samples (labelled as A, B and C) related to extracted materials presented as average bars in Figure 6.

**Figure 6:**
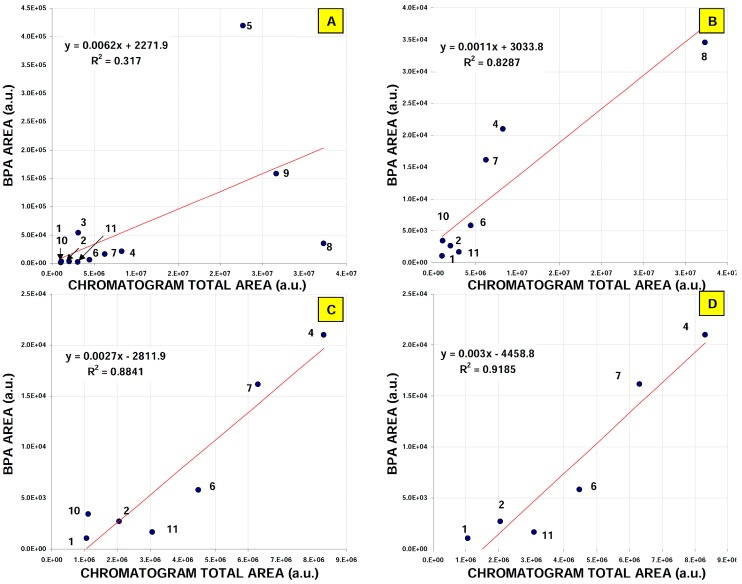
Correlation observed between SPE extracts contents (the total peaks area on UV-DAD chromatograms) and the bisphenol A peak area for each extracted material. Sample labelling: (**A**). all tested materials; (**B**). without samples 3, 5 and 9; (**C**).without samples 3, 5, 9 and 8; (**D**). without samples 3, 5, 9, 8 and 10. Dots numbers corresponds to material types listed in Table 5 and Table 6.

Quantitative data (Table 6) has revealed a possible high level of bisphenol A that may be emitted from rice bags in comparison with tap water samples, even if the overall area of chromatographic profiles for these samples were similar (Figure 4). Generally, all of the materials investigated can be a source of bisphenol A and related micropollutants, particularly wet wipes, plastic bags and fish baits. Within each material type, a high variability in the level of total organic matrix is observed (Figure 5). There is no correlation between the bisphenol A level and the total organic matrix eluted from SPE tubes and detected by UV-Vis detector for all samples investigated (Figure 6A). Nevertheless, such a correlation can be significant for selected materials (Figure 6B–D). This observation may be applied to the design of low-resolution microfluidic systems (e.g., paper based microfluidic devices), enabling fast screening for the presence of such compounds in polymers related samples. Simply put, as the first screening step, the SPE extracts should be analyzed using low-resolution microfluidic devices. All samples characterized by high contents of organic matrix should be then quantified by more specific and sensitive HPLC or GC protocols.

It should be noted that the extracted mass of bisphenol A from fish bait material (Table 6), may cause a real pollution problem for small water ecosystems. For example, a typical lake with high anthropogenic pressure from the angling community at the Middle Pomerania area close to the Koszalin City (Poland) e.g., Lake MorskieOko (N 54.079093, E 16.472463; Figure S3; Supporting Information) with a water volume of 4 × 10^5^ m^3^ (lake diameter 212 m and depth 23 m) can be compromised by high number of fish baits, which were lost on underwater hooks. This situation is realistic due to frequent reports from divers who saw a number of fish baits on e.g., underwater tree residues in this lake. For our estimations, the following assumptions were made: the possible amount of plastic containing flexible fish baits that can be lost on underwater hooks over e.g., 10 years ≈ 1000; this corresponds to the total mass of 10 g of bisphenol, which may be emitted from such number of fish baits (considering that average mass of each fish bait = 5 g). Based on these assumptions a pollution level close to 25 ng/L may be expected in this lake. It should be mentioned that even if the flexible fish baits analyzed are made of silicone related materials, for storage purposes, they are lubricated with unknown oils and packed in various plastic containers. This allows for the uncontrolled diffusion of plasticizers (like bisphenol A) to fish baits, and then to the water ecosystem.

## 3. Materials and Methods

### 3.1. Materials

As the chromatographic standards following reagents were used: bisphenol B; (ChemCruz, Dallas, TX, USA), bisphenol A, BP, Z, AP, C, E, F, FL and S (Sigma-Aldrich, St. Louis, MO, USA) as well as 7,8-dimethoxyflavone (internal standard; Sigma-Aldrich, Steinheim, Germany) and sodium nitrate (dead time marker, POCh Gliwice, Gliwice, Poland). HPLC mobile phases additives, namely β-cyclodextrin and 2-hydroxypropyl-β-cyclodextrin were purchased from Merk (Darmstadt, Germany) and Sigma-Aldrich (Steinheim, Germany), respectively. Organic liquids (LiChrosolv acetonitrile 99% and LiChrosolv methanol 99.8%), which were used as the solvents for chromatographic standards and mobile phases (HPLC) and SPE cleaning/elution solvents preparation, were obtained from Merck (Darmstadt, Germany). Binary chromatographic mobile phases were prepared using freshly distilled tap water.

As the samples number of daily used products were investigated including rice bags, plastic bags, cloths, sanitary towels and fish baits. These commercially available products were purchased in general stores in Koszalin City (Poland) and are listed in Table 5.

Environmental samples (700 mL of treated wastewater, approximately) were collected (five times: 2018_04_16–18, 2018_04_25–26) from chamber of the secondary settling tank placed in the Jamno Sewage Treatment Plant, located close to Koszalin area (N 54° 14.196′ E 16° 9.528′). Wastewater samples were immediately processed by SPE isolation, pre-purification and concentration protocol.

### 3.2. Instrumentation and Analytical Protocols

#### 3.2.1. Solid-Phase Extraction (SPE)

For our research, solid phase extraction was the central part of samples analysis. This key step was performed using SPE Supelclean TM LC-18 tubes, (5 mL, 0.5 g columns obtained from Supelco, Bellefonte, PA, USA) and a SPE vacuum chamber (Supelco, Bellefonte, PA, USA) connected to a N86 vacuum pump. KN 18 KNF (NuebergerLaboport, Freiburg, Germany). The core protocol and the sequence of SPE procedure was based on the analytical procedures invented previously by our research teams and designed for purification/concentration of wide range of polar compounds from liquid samples [11,12,13,26,53]. Briefly, the SPE columns were conditioned using 5 × 1 mL of 100% methanol and 5 × 1 mL methanol/water (1%, *v*/*v*). Samples (250 mL) were passed through the SPE columns and then purified with a cleaning mixture (5 × 1 mL methanol/water, 30%, *v*/*v*). The target compounds were eluted with four portions of 0.5 mL of 100% methanol and obtained liquid was evaporated at room temperature in a Savant SPD121P vacuum centrifuge (Thermo Electron Corporation, Milford, MA, USA), which was connected to a cold trap (Refigerated Vapor Traps RVT 4104, Asheville, NC, USA) and the Thermo Savant VLP80 oil vacuum pump, model RV3 (Thermosavant Instruments Inc., Holbrook, NY, USA). The dry residue was dissolved in 100 μL of mobile phase without the addition of cyclodextrins (acetonitrile/ water, 35%, *v*/*v*).

For recovery studies, the sample of 1000 mL of distilled water was spiked with 1 μg mass of internal standard and 0.1 μg mass of each bisphenol investigated (11 target components). Next, 100 μL (35%, *v*/*v*, acetonitrile/water) of stock solution containing 1 μg/mL of bisphenols mixture and 10 μg/mL internal standard was added to 1000 mL volume of the sample. Volume of 1000 μL of stock solution was prepared by mixing of 10 μL × 11 (= 110 μL) of each bisphenol (at concentration of 100 μg/mL in 35%, *v*/*v*, acetonitrile/water), 10 μL of internal standard at concentration of 1 mg/mL in ethanol and 880 μL of solvent (35%, *v*/*v*, acetonitrile/water). Then SPE procedure was applied. Final sample for HPLC determination was reconstituted in 100 μL of acetonitrile/water (35%, *v*/*v*).

For screening studies (involving chromatographic profiles analysis detected by DAD-UV-Vis detector concerning daily used products and wastewater samples), the following protocol was applied: to 250 mL of tap water, 2 g of solid material was added. The quantities of various solid materials (listed above) were investigated. For the selected food samples (e.g., rise packaging), samples were boiled for 15 min. at 100 °C, cooled to room temperature, and spiked with 0.25 μg of internal standard (IS volume of 25 μL at concentration of 10 μg/mL in 35%, *v*/*v*, acetonitrile/water). In the case of wastewater, a 250 mL sample was mixed with IS, as above. Wastewater chromatographic profiles were collected for raw material and after heating of the sample in 100 °C as described above. Then, SPE procedure was applied. Final sample for HPLC determination was reconstituted in 100 μL of acetonitrile/water (35%, *v*/*v*).

#### 3.2.2. Chromatography (HPLC)

Optimization of column chromatographic separation of bisphenols standards and real samples was conducted using hardware setup described previously for selected naphthalene derivatives [25]. Basically, we used a chromatographic system where column temperature was controlled by foam insulated water jacket connected to circulating thermostat (Nestlab RTE7; product of Thermo Electron Corporation, Newington, NH, USA). HPLC system consisted isocratic pump (LC-10ADvp), injector (Rheodyne 7725i, Rohner Park, CA, USA) with 20μL loop, a SPD-M20A photodiode array detector (DAD) and a computer system for data acquisition with software LC Solution (version 1.21 SP1; 2002–2005), which was product of Shimadzu (Suzhou New District, Jiangsu, China).

Due to solubility limitation, stock solutions of bisphenols (1 mg/mL) were prepared in ethanol. HPLC separation was carried out using isocratic system and the mobile phase flow of 1 mL/min. The hold up time (*t_o_*; dead time) of column chromatographic system was monitored each day using sodium nitrate marker (10 μg/mL) dissolved in the mobile phase without cyclodextrins additive (acetonitrile/water, 35%, *v*/*v*).

## 4. Conclusions

### 4.1. Detailed Conclusions to Part 2.1. (Optimization of Bisphenols Separation)

High recovery of bisphenols clearly indicates that the previously optimized by our research teams the SPE protocol can be successfully used to determine these compounds from liquid samples. Particularly, compositions and volumes of cleaning and eluting solvents (primarily targeting low-molecular mass chemicals with polarities ranging from estetrol to progesterone) can be applied for bisphenols extraction, purification and pre-concentration.

Chromatographic data revealed that both β-CD and its hydroxypropyl derivative strongly interact with selected bisphenols. This is contrary to the steroids and PAHs molecules investigated previously, where a strong interaction with β-cyclodextrin was observed.

The proposed SPE extraction and chromatographic determination are simple, non-expensive, and are based on biodegradable materials; therefore, they can be considered a green chemistry method for the efficient fractionation, extraction and separation of bisphenols from complex environmental and food related samples.

The addition of given macrocycles, namely native β-cyclodextrin and its hydroxypropyl derivative, to the liquid phase significantly changes the retention behavior of the target (guest) molecules including polycyclic aromatic hydrocarbons (naphthalene, its methyl derivatives and acenaphthenol optical isomers), as well as a battery of selected bisphenols (A, B, C, E, F, S, Z, AF, AP, BP, FL) in the liquid phase, both under static (solutions) and dynamic (chromatographic separation) conditions. It has been documented that this phenomenon is more visible at sub-ambient temperatures (temperatures ranging from 5 to 20 °C), similar to different classes of low-molecular mass compounds investigated previously (e.g., steroid hormones acting as endocrine modulators). However, experimental data revealed that supramolecular interactions at elevated temperatures (25–50 °C) are also possible for selected host molecules (bisphenols). The column chromatographic experiment focusing on the separation efficiency of selected bisphenols in the presence of macrocyclic additives clearly indicated that such modifiers can significantly improve analysis times and the selectivity of the isocratic system at the given temperature for simultaneous determination of various bisphenols mixtures. An optimized chromatographic protocol based on octadecylsilane column and acetonitrile/water binary mobile phase can be applied for fast and non-expensive quantification of target compounds involving green chemistry protocols. The proposed quantification protocol, due to its simplicity, may be applied for highly selective monitoring of micropollutants during technological wastewater treatment processes.

### 4.2. Detailed Conclusions to Part 2.2. (Real Samples Analysis)

The described extraction and quantification protocols are capable of rapidly screening bisphenols and related low-molecular mass micropollutants fraction from various complex materials. Quantitative data has revealed the problem of bisphenol A being released into the environment, and also in the case of sewage water produced by wastewater treatment processes. It has been proven that: (i) some food products may emit the high levels of low-molecular mass compound fractions (with polarity from estetrol to progesterone) during the cooking process, and (ii) that common fish baits may cause real environmental pollution of freshwater ecosystems. Particularly, it has been demonstrated that a whole range of low-molecular mass compounds which may be detected using UV-Vis detector can easily be emitted from various in daily use products. This issue of the presence of micropollutants in treated wastewater, water ecosystems and plastic waste utilization via technological wastewater treatment processes, especially in terms of pollutants originating from microplastic which act as endocrine disrupters, must be seriously addressed.

## Figures and Tables

**Table 1:** Values of chromatographic retention factor (*k*) of analytes separated on a 10 cm long LC-18 column at various temperatures and using mobile acetonitrile/water (35%, *v*/*v*) unmodified (**A**) and modified phases β-cyclodextrin (**B**) and its hydroxypropyl derivative (**C**) at a concentration of 10 mM. The substance numbers correspond to the order of the analytes given in the Figure S1; Supporting Information.

Analyte	Separation Temperature [°C]				
	10	20	30	40	50
*k* values.					
**A** (unmodifed mobile phase)					
Bisphenol S (1)	2.863	2.162	1.959	1.629	1.394
Bisphenol F (2)	6.383	4.889	4.420	3.305	3.105
Bisphenol E (3)	9.483	7.428	6.487	5.165	4.592
Bisphenol A (4)	13.837	10.665	9.398	7.365	6.523
7,8-Dimethoxyflavone (12)	22.674	18.213	17.051	14.287	13.077
Bisphenol B (5)	26.150	19.277	16.722	12.756	11.187
Bisphenol C (6)	40.741	29.965	26.902	20.321	17.829
Bisphenol AP (7)	48.169	33.228	29.417	22.246	18.394
Bisphenol BP (8)	50.195	34.221	29.174	21.318	18.509
Bisphenol Z (11)	53.030	37.737	32.982	24.294	21.462
Bisphenol AF (9)	52.820	38.002	32.990	24.858	20.700
Bisphenol FL (10)	100.303	67.296	57.400	41.380	34.518
**B** (β-cyclodextrin in mobile phase)					
Bisphenol S	1.770	1.585	1.412	1.295	1.188
Bisphenol F	2.595	2.474	2.313	2.166	2.028
Bisphenol E	2.749	2.736	2.679	2.617	2.518
Bisphenol A	3.048	3.041	2.980	2.854	2.722
7,8-Dimethoxyflavone	16.462	15.147	13.772	12.419	11.084
Bisphenol B	4.687	4.976	5.195	5.237	5.170
Bisphenol C	18.422	17.453	16.363	14.748	13.050
Bisphenol AP	24.576	22.071	18.898	16.030	13.434
Bisphenol BP	24.504	21.709	18.851	16.048	13.433
Bisphenol Z	4.715	5.161	5.540	5.825	5.966
Bisphenol AF	28.970	25.442	21.908	18.471	15.443
Bisphenol FL	58.406	49.536	40.997	33.466	26.676
**C** (2HP-β-cyclodextrin in mobile phase)					
Bisphenol S	1.656	1.456	1.305	1.157	1.086
Bisphenol F	2.289	2.129	2.010	1.908	1.793
Bisphenol E	2.302	2.293	2.273	2.214	2.223
Bisphenol A	2.485	2.605	2.685	2.752	2.746
7,8-Dimethoxyflavone	14.581	13.315	12.066	10.968	9.841
Bisphenol B	4.208	4.413	4.526	4.634	4.511
Bisphenol C	14.638	14.328	13.557	12.680	11.253
Bisphenol AP	16.392	15.127	13.562	11.920	10.716
Bisphenol BP	16.507	15.161	13.663	11.913	10.583
Bisphenol Z	4.293	4.640	4.941	5.112	5.271
Bisphenol AF	21.881	19.333	16.793	14.742	12.395
Bisphenol FL	46.732	39.875	33.112	27.068	22.372

**Table 2:** Values of non-linear regression coefficients and determination coefficient (*r*^2^) for the equation in the form ln*k* = ax^2^ + bx+ c (where x = 1000/T) for substances chromatographed on a 10 cm LC-18 column and mobile acetonitrile/water phases (35%, *v*/*v*) without a macrocyclic modifier and with the addition of β-cyclodextrin and hydroxypropyl-β-cyclodextrin, calculated on the basis of the data presented in Table 1.

Analyte	Unmodified Mobile Phase				10mM β-Cyklodextrin				10mM 2 HP-β-Cyklodekstrin			
	*a*	*b*	*c*	*r* ^2^	*a*	*b*	*c*	*r* ^2^	*a*	*b*	*c*	*r* ^2^
Bisphenol S	0.58	−2.23	1.85	0.986	0.18	−0.25	−0.73	0.999	0.40	−1.69	1.44	0.997
Bisphenol F	0.77	−3.45	4.38	0.974	−0.39	3.17	−5.35	0.999	−0.0006	0.55	−1.11	0.998
Bisphenol E	0.46	−1.39	1.42	0.993	−0.47	3.34	−4.88	0.998	−0.057	0.47	−0.12	0.863
Bisphenol A	0.44	−1.25	1.45	0.990	−0.59	3.64	−4.5	0.994	−0.55	3.39	−4.24	0.994
Bisphenol B	0.73	−2.94	4.46	0.990	−1.00	6.43	−8.63	0.995	−0.84	5.35	−7.07	0.962
Bisphenol C	0.53	−1.69	2.96	0.983	−1.28	9.28	−13.8	0.999	−1.41	9.91	−14.78	0.996
Bisphenol AP	0.65	−2.23	3.52	0.984	−1.32	10.1	−16.14	0.999	−0.755	5.99	−8.95	0.998
Bisphenol BP	1.31	−6.42	10.2	0.988	−1.21	9.40	−14.9	1	−0.92	7.15	−10.93	0.998
Bisphenol Z	0.95	−4.26	7.1	0.985	−0.75	4.42	−4.72	0.999	−0.59	3.50	−3.45	0.999
Bisphenol AF	0.31	0.05	−0.01	0.990	−1.15	9.08	−14.3	1	−0.90	7.28	−11.35	0.999
Bisphenol FL	0.92	−3.7	6.16	0.988	−1.36	10.8	−17.15	1	−1.00	8.36	−13.15	0.999

**Table 3:** Retention factor ratios (*k*_0mMCD_/*k*_10mMCD_) reflecting host-guest interaction intensity calculated from data presented in Table 3 for β-cyclodextrin (**A**) and 2-hydroxypropyl-β-cyclodextrin (**B**); Target components highlighted in red were selected for degradation experiment with duckweed under different conditions *(Chapter 4.3.2.3)*.

Analyte	Separation Temperature °C				
	10	20	30	40	50
A: *k*_0mMCD_/*k*_10mMCD_ ratio values for β-cyclodextrin					
Bisphenol A	5.02	3.86	3.07	2.51	2.11
Bisphenol AF	1.78	1.57	1.45	1.37	1.33
Bisphenol AP	1.89	1.64	1.49	1.40	1.36
Bisphenol B	5.44	4.03	3.13	2.53	2.11
Bisphenol BP	1.96	1.68	1.50	1.39	1.33
Bisphenol C	2.16	1.80	1.58	1.43	1.34
Bisphenol E	3.40	2.80	2.36	2.04	1.79
Bisphenol F	2.41	2.08	1.83	1.64	1.48
Bisphenol FL	1.65	1.46	1.34	1.28	1.26
Bisphenol S	1.57	1.45	1.35	1.26	1.18
Bisphenol Z	10.89	7.73	5.72	4.39	3.48
B: *k*_0mMCD_/k_10mMCD_ ratio values for hydroxypropyl β-cyclodextrin					
Bisphenol A	5.47	4.24	3.38	2.78	2.33
Bisphenol AF	2.36	2.07	1.87	1.74	1.65
Bisphenol AP	2.82	2.39	2.09	1.87	1.71
Bisphenol B	6.07	4.56	3.56	2.89	2.41
Bisphenol BP	2.90	2.42	2.08	1.85	1.69
Bisphenol C	2.72	2.20	1.88	1.68	1.55
Bisphenol E	4.04	3.34	2.80	2.38	2.04
Bisphenol F	2.74	2.39	2.11	1.87	1.67
Bisphenol FL	2.06	1.82	1.66	1.57	1.51
Bisphenol S	1.67	1.58	1.48	1.38	1.29
Bisphenol Z	11.93	8.60	6.43	4.97	3.95

**Table 4:** Recovery values of bisphenols and internal standard (*n* = 5) at concentration corresponding to 100 ng/L and 1 µg/L (for each bisphenols and IS, respectively) of water sample for the SPE procedure tested.

Analyte	Average Recovery (%)	STD	CV%
BPS	28.91	5.57	19.28
BPF	95.22	4.07	4.27
BPE	97.27	0.91	0.93
BPA	213.64	21.10	9.88
BPB	96.14	3.38	3.52
BPZ	101.14	8.44	8.35
BPC	93.02	11.38	12.24
BPAP + BPBP	77.69	12.33	15.87
BPAF	159.13	72.01	45.25
BPFL	92.95	3.44	3.70
7,8-DMF	94.35	6.96	7.37

**Table 5:** List of samples and materials analyzed by SPE/HPLC/DAD-UV-Vis protocol.

No	Samples and Materials
1	Tap water boiled (250 mL)
2	Rice bags (2 g)
3	Plastic bags (2 g)
4	Cloth (2 g)
5	Fish baits (2 g)
6	Sanitary towels (2 g)
7	Wet wipes (2 g)
8	Boiled purified sewage (250 mL)
9	Raw purified sewage (250 mL)
10	Distilled water (250 mL)
11	Raw tap water (250 mL)

**Table 6:** Quantitative data (values of peak heights and bisphenol A/internal standard ratio) for all samples investigated.

Sample Source	BPA (Peak Heights; a.u.)	IS (Peak Heights; a.u.)	BPA/IS Ratio *{Concentration µg/L}*
Sample Type: Boiled Tap Water (**1**)			
(A) (*n* = 2)	865.0	4053.0	0.21
(B) (*n* = 2)	1307.5	2749.5	0.47
(C) (*n* = 2)	1039.0	4118.5	0.25
AVG	1070.5	3640.3	**0.31** ***{12.9}***
STD	222.9	772.2	
CV%	20.8	21.2	
Sample Type: Rice Bags (**2**)			
(A) (*n* = 2)	1604.5	4225.0	0.38
(B) (*n* = 2)	1761.0	4190.5	0.42
(C) (*n* = 2)	4657.5	5290.0	0.88
AVG	2674.3	4568.5	**0.56** ***{23.3}***
STD	1719.3	625.1	
CV%	64.3	13.7	
Sample Type: Plastic Bags (**3**)			
(A) (*n* = 2)	257.0	1627.0	0.16
(B) (*n* = 2)	155957.0	7043.0	22.14
(C) (*n* = 2)	3730.5	3068.5	1.22
AVG	53314.8	3912.8	**13.63** ***{567,5}***
STD	88907.7	2805.0	
CV%	166.8	71.7	
Sample Type: Cloth (**4**)			
(A) (*n* = 2)	54927.5	4747.0	11.57
(B) (*n* = 2)	4265.5	7043.0	0.61
(C) (*n* = 2)	3730.5	3068.5	1.22
AVG	20974.5	4952.8	**4.23** ***{176.8}***
STD	29405.4	1995.2	
CV%	140.2	40.3	
Sample Type: Fish Baits (**5**)			
(A) (*n* = 2)	5953.5	2362.0	2.52
(B) (*n* = 2)	248460.5	4492.5	55.31
(C) (*n* = 2)	1003649.5	4882.0	205.58
AVG	419354.5	3912.2	**107.19** ***{4466.2}***
STD	520339.2	1356.5	
CV%	124.1	34.7	
Sample Type: Sanitary Towels (**6**)			
(A) (*n* = 2)	7370.0	522.5	14.11
(B) (*n* = 2)	705.5	6736.5	0.10
(C) (*n* = 2)	9342.0	4496.5	2.08
AVG	5805.8	3918.5	**1.48** ***{61.7}***
STD	4525.7	3147.1	
CV%	78.0	80.3	
Sample Type: Wet Wipes (**7**)			
(A) (*n* = 2)	13330.5	4124.0	3.23
(B) (*n* = 2)	11834.5	2527.5	4.68
(C) (*n* = 2)	23285.5	3303.5	7.05
AVG	16150.2	3318.3	**4.87** ***{203}***
STD	6224.5	798.4	
CV%	38.5	24.1	
Sample Type: Boiled Purified Sewage (**8**)			
(A) (*n* = 2)	61888.5	4598.0	13.46
(B) (*n* = 2)	362477.0	3326.5	108.97
(C) (*n* = 2)	50848.0	3326.5	15.29
AVG	158404.5	3750.3	**42.24** ***{1760.8}***
STD	176818.2	734.1	
CV%	111.6	19.6	
Sample Type: Raw Purified Sewage (**9**)			
(A) (*n* = 2)	35839.0	2907.5	12.33
(B) (*n* = 2)	37057.0	4032.0	9.19
(C) (*n* = 2)	30623.0	3700.0	8.28
AVG	34506.3	3546.5	**9.73** ***{405.4}***
STD	3417.8	577.8	
CV%	9.9	16.3	
Sample Type: Distilled Water (**10**)			
(A) (*n* = 2)	3233.0	24947.0	0.13
(B) (*n* = 2)	3640.0	27988.0	0.13
(C) (*n* = 2)	3325.0	26221.0	0.13
AVG	3399.3	26385.3	**0.13** ***{5.41}***
STD	213.4	1527.1	
CV%	9.9	16.3	
Sample Type: Raw Tap Water (**11**)			
(A) (*n*=2)	1896.0	4193.0	0.45
(B) (*n*=2)	1695.0	3974.0	0.43
(C) (*n*=2)	1400.0	4257.0	0.33
AVG	1663.7	4141.3	**0.40** ***{16.7}***
STD	249.5	148.4	
CV%	15.0	3.6	

## Supplementary Materials

The following are available online. Figure S1: Chemical structures of selected bisphenols and internal standard substance (7,8-dimethoxyflavone) for chromatographic analysis of target analytes. Figure S2: HPLC chromatograms of SPE extracts of environmental samples and recorded at analytical wavelength = 280 nm. Figure S3: Geographical location and bathymetric intersection through “Jezioro Morskie Oko” lobelia lake.
