# Supplementary material for: Analysis of Selected Endocrine Disrupters Fraction Including Bisphenols Extracted from Daily Products, Food Packaging and Treated Wastewater Using Optimized Solid-Phase Extraction and Temperature-Dependent Inclusion Chromatography
Source: Molecules. 2019 Apr 2;24(7):1285. doi: 10.3390/molecules24071285 (PMC6479962; doi:10.3390/molecules24071285)
Supplement: Supplementary file 1 [file molecules-24-01285-s001.pdf]

Supporting Information

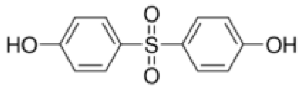

(1) Bisphenol S
Bis(4-hydroxyphenyl) sulfone

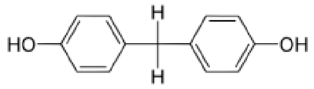

(2) Bisphenol F
Bis(4-hydroxyphenyl) methane

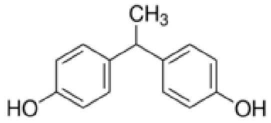

(3) Bisphenol E
1,1-Bis(4-hydroxyphenyl) ethane

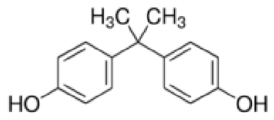

(4) Bisphenol A
2,2-Bis(4-hydroxyphenyl) propane

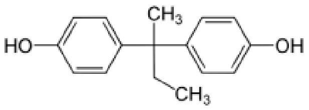

(5) Bisphenol B
2,2-Bis(4-hydroxyphenyl) butane

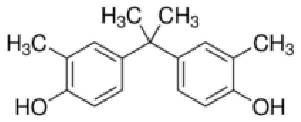

(6) Bisphenol C
2,2-Bis(3-methyl-4-hydroxyphenyl) propane

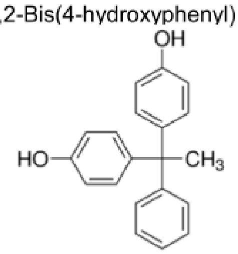

(7) Bisphenol AP
1,1-Bis(4-hydroxyphenyl)-1 phenyl-ethane

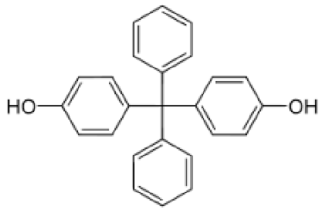

(8) Bisphenol BP
Bis-(4-hydroxyphenyl) diphenylmethane

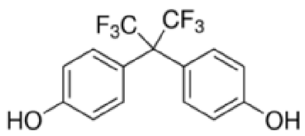

(9) Bisphenol AF
2,2-Bis(4-hydroxyphenyl) hexafluoropropane

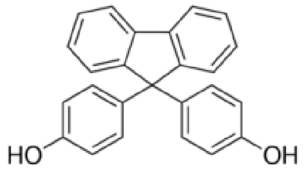

(10) Bisphenol FL
4,4'-(9-fluorenylidene) diphenol

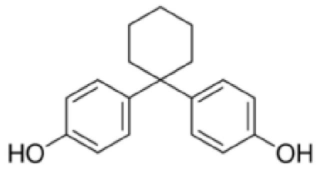

(11) Bisphenol Z
1,1-Bis(4-hydroxyphenyl)-cyclohexane

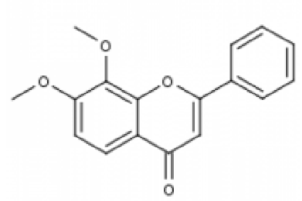

(12) 7,8 Dimethoxyflavone

Figure S1. Chemical structures of selected bisphenols and internal standard substance (7,8-dimethoxyflavone) for chromatographic analysis of target analytes.

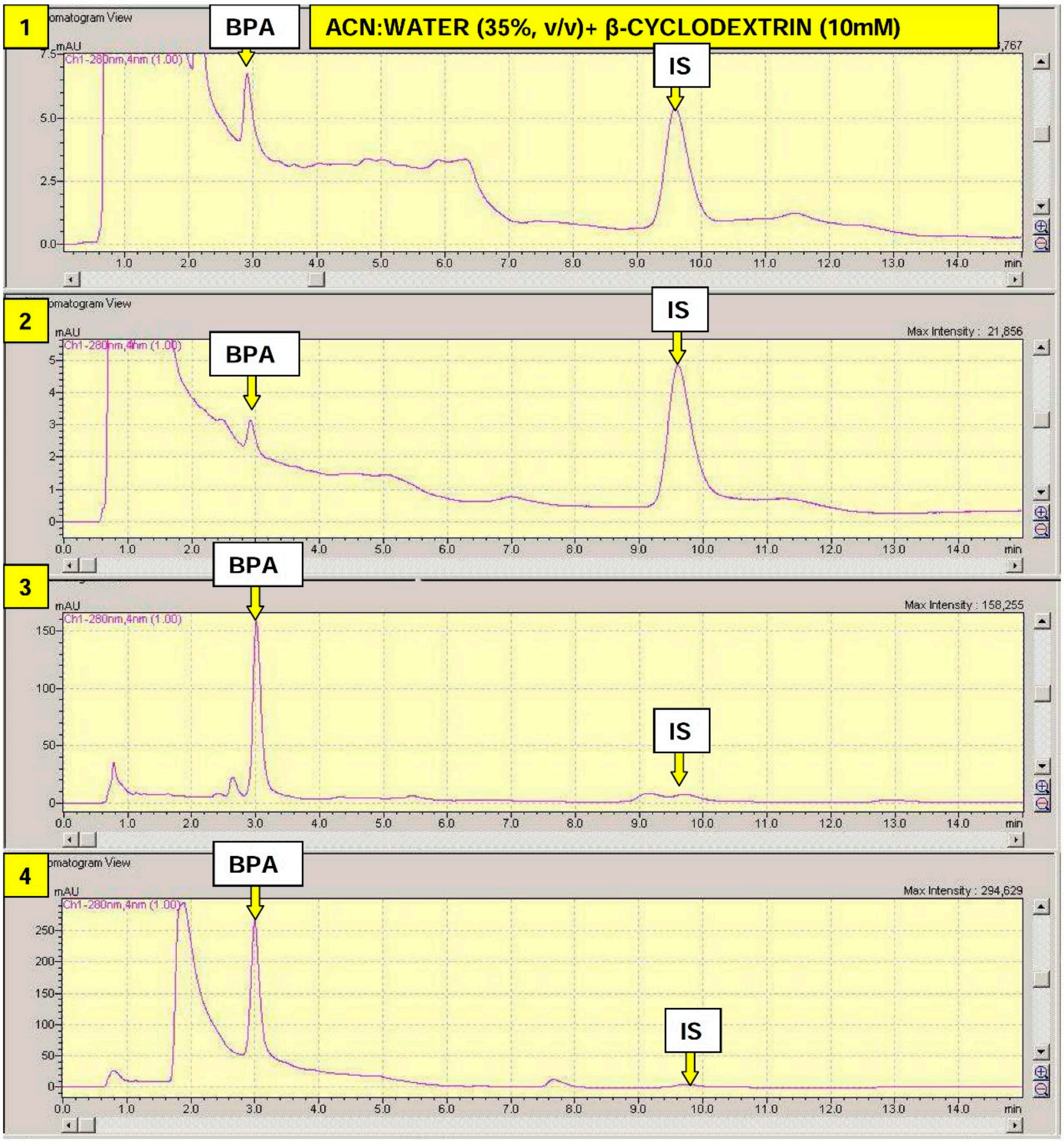
1
BPA
ACN:WATER (35%, v/v)+ β-CYCLODEXTRIN (10mM)
IS
2
BPA
IS
Max Intensity : 21,856
3
BPA
IS
Max Intensity : 158,255
4
BPA
IS
Max Intensity : 294,629
mAU
min

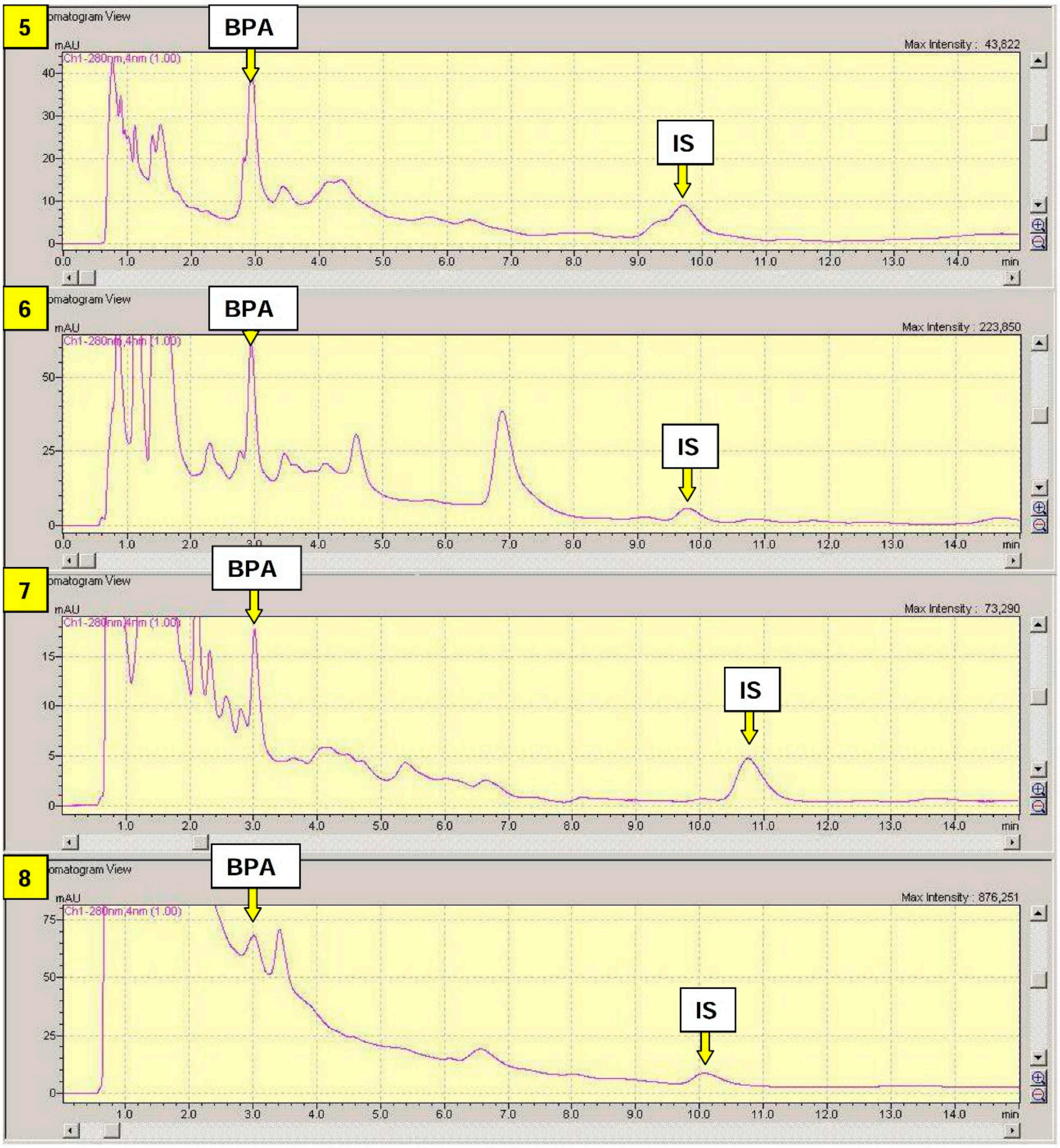
5
omatogram View
BPA
IS
mAU
Max Intensity : 43,822
Ch1-280nm,4nm (1.00)
min
6
omatogram View
BPA
IS
mAU
Max Intensity : 223,850
Ch1-280nm,4nm (1.00)
min
7
omatogram View
BPA
IS
mAU
Max Intensity : 73,290
Ch1-280nm,4nm (1.00)
min
8
omatogram View
BPA
IS
mAU
Max Intensity : 876,251
Ch1-280nm,4nm (1.00)
min

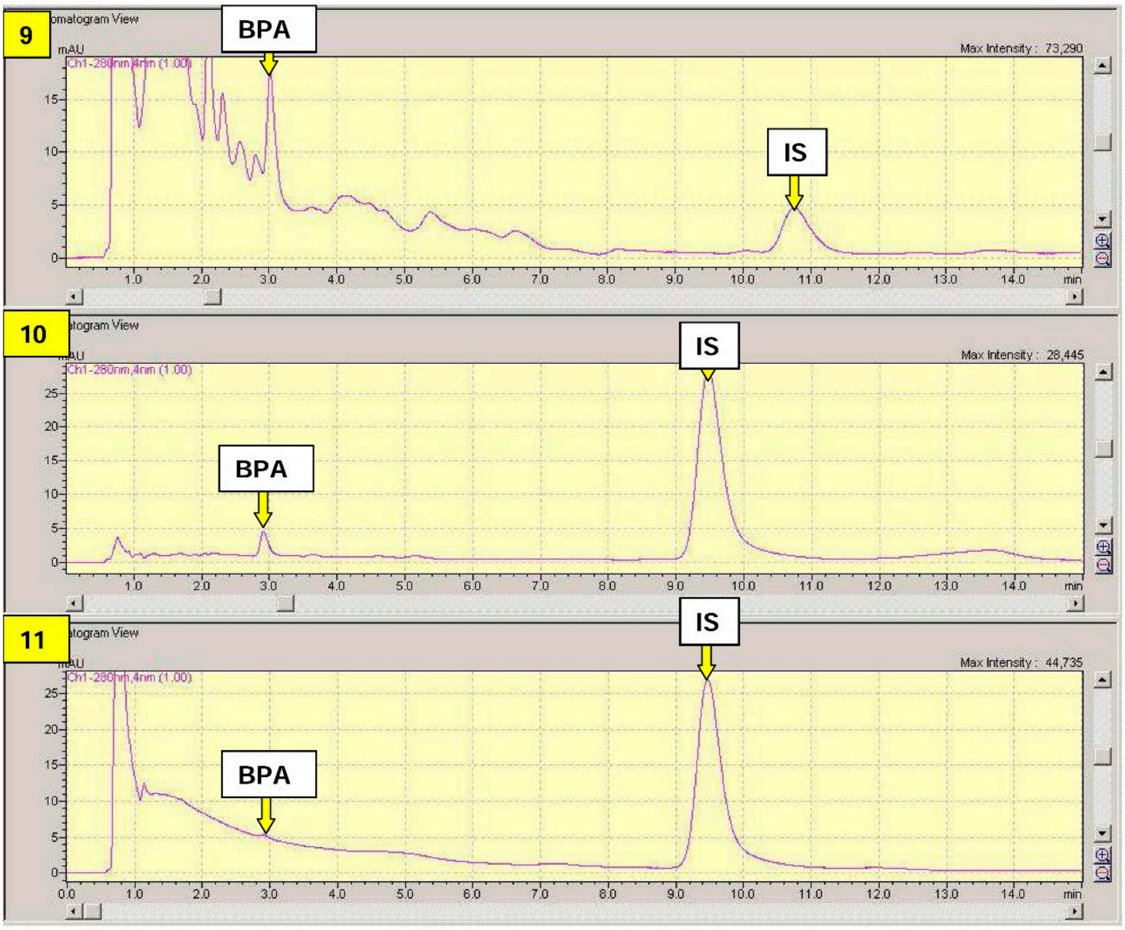

Figure S2. HPLC chromatograms of SPE extracts of environmental samples and recorded at analytical wavelength = 280 nm. Sample labels: **1** Boiled tap water and 7,8 dimethoxyflavone (IS), **2** Tap water and rice bags and 7,8 dimethoxyflavone, **3** Tap water and plastic (braeafast bags) and 7,8 dimethoxtflavone, **4** Tap water and cleaning cloths and 7,8 diethoxyflavone, **5** tap water and fish bait and 7,8 dimethoxyflavone, **6** tap water and sanitary towels and 7,8 dimethoxyflavone, **7** tap water and wet wipes and 7,8 dimethoxyflavone, **8** boiled purified sewage and 7,8 dimethoxyflavone. **9** Raw purified sewage and 7,8 dimethoxyflavone, **10** distilled water and 7,8 dimethoxyflavone, **11** Raw tap water and7,8 dimethoxyflavone. Bisphenol A (BPA) peak is marked on all chromatograms.

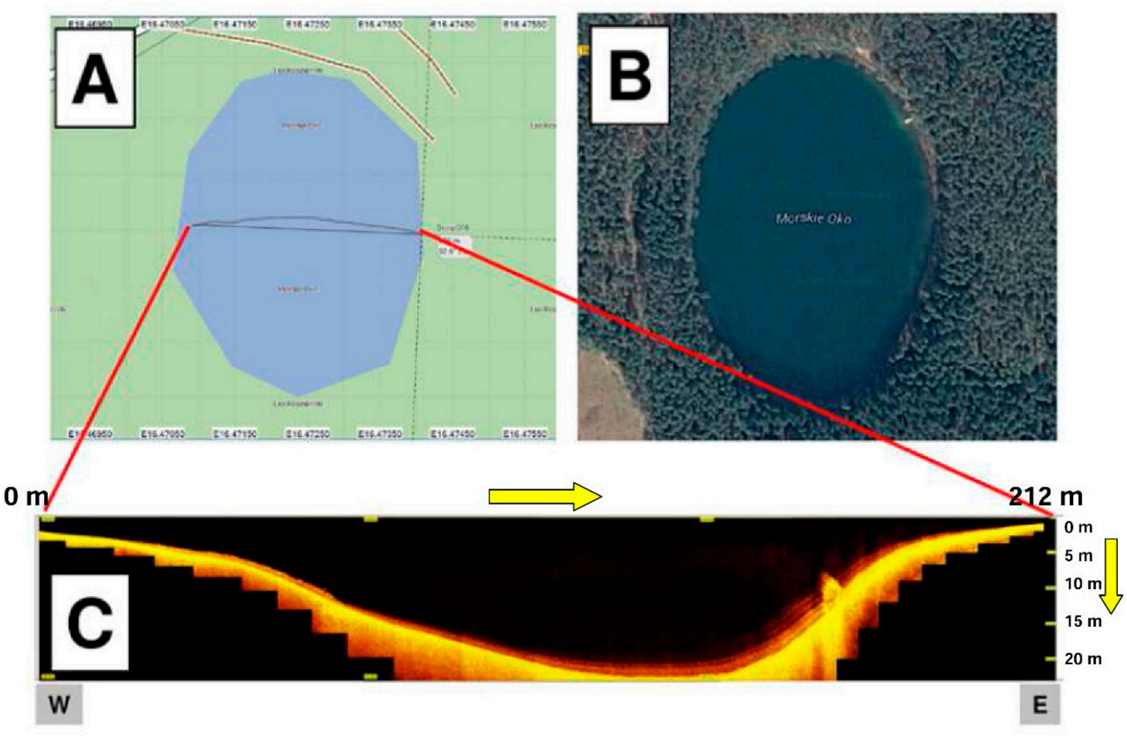

Figure S3. Geographical location (A,B maps provided by Garmin and GoogleMaps, respectively) and bathymetric intersection (22.03.2015) through JezioroMorskieOko lobelia lake. Depth profile was made using Garmin echoMAP 50dv marine chartplotter/sonar equipped with transducer GT 20 (77/200/455 kHz) working with high frequency 455 kHz (DownVü mode); lake cross-section was visualized using Home Port Ver. 2.2.1.0 2009-2015 Garmin Ltd. freeware; Copyright @ 2015 Paweł K. Zarzycki with permission.
